# Editorial: Food pathogens and antimicrobial resistance

**DOI:** 10.3389/fmicb.2023.1243769

**Published:** 2023-07-04

**Authors:** Yang Wang, Jianmin Zhang, Zhong Peng, Xiangru Wang, Paul Plummer

**Affiliations:** ^1^College of Animal Science and Technology, Henan University of Science and Technology, Luoyang, China; ^2^College of Veterinary Medicine, South China Agricultural University, Guangzhou, China; ^3^College of Animal Science and Technology, Huazhong Agriculture University, Wuhan, China; ^4^College of Veterinary Medicine, Iowa State University, Ames, IA, United States

**Keywords:** antimicrobial resistance, foodborne pathogens, microorganisms, resistance gene, genetic information

The issue of antimicrobial resistance (AMR) of microorganisms is prevalent worldwide (Neely and Holder, 1999). The overuse of antimicrobials in agricultural animals for food production has been proposed as the leading cause of the proliferation of antimicrobial-resistant pathogens (Minarini et al., 2020). Raw meat, cooked food products, and raw milk are often contaminated by food-borne pathogens, many of which are resistant to a variety of antimicrobials (Chao et al., 2007). The exchange of genetic information among food-borne pathogens is a major factor in the development of antimicrobial resistance. Therefore, monitoring antimicrobial-resistant at different nodes in the food chain is particularly important to understand the spread of antimicrobial-resistant (Di Ciccio, 2021).

In this Research Topic, nine original research articles and one review have been published. Hu et al. comprehensively reviewed the characteristics, epidemiology, pathogenic mechanisms, zoonotic potential, antimicrobial resistance, diagnosis, alternative control measures, and vaccine development of avian pathogenic *E. coli* (APEC).

Research has shown that intestinal microbiota plays a critical role in maintaining the integrity of gut barrier, and specific microorganisms in digestive tracts can aid in the treatment of gastrointestinal diseases, thereby reducing the reliance of antimicrobials and other drugs (Gresse et al., 2017). Using a model of Porcine epidemic diarrhea virus (PEDV)-infected LC and Large-white piglets established by Li et al., the authors analyzed differences in intestinal microbial diversity, community composition, and intestinal metabolites between PEDV-infected and healthy control piglets. This study offers a theoretical foundation for utilizing intestinal core microorganisms in the digestive system of PEDV-infected pigs, to address the issue of piglet diarrhea that arises from PEDV infection.

Bacterial AMR is usually regulated by genes. The research conducted by Tang et al. demonstrated that the plasmid-borne *cfr* gene facilitates multidrug resistance (MDR), which subsequently leads to *cfr*-positive *E. coli* exhibiting MDR. Moreover, the study revealed that *cfr* can form a circular intermediate of IS26-*cfr* during transmission, confirming IS26's significant role in the dissemination of the multidrug resistance gene *cfr*.

In the study of Chen et al., the genes that play a key role in multiple drug resistance in Bacillus cereus are *hblA, hblC, hblD, nheA*, and *nheB*. Lai et al.'s research indicate that the AMR of *Salmonella* is primarily attributed to the significant impact of the plasmid-mediated quinolone resistance gene *pmqr*, the β-lactam resistance gene *bla*_Tm_, and the mutation of the quinolone resistance-determination region (QRDR) that contains the *pmqr* gene.

Ji et al. sequenced the complete genome of 322 *Listeria monocytogenes* strains isolated from food and discovered that these strains carry the drug resistance genes *aacA4, etM, Tets*, and *dfrG*. Furthermore, the researchers discovered a novel premature stop codon in the *inlA* gene, leading to a better comprehension of the genomic diversity of *Listeria monocytogenes*.

Buberg et al. studied the survival rate, colonization characteristics and conjugation ability of ESC-resistant *E. coli* isolates through a static *in vitro* digestion model (INFOGEST). The findings demonstrate that the strains are capable of surviving and reproducing within the *in vitro* digestion model. Furthermore, the strains exhibit the capability to adhere to and invade colon cells post-digestion, with a higher degree of adhesion to colon cells observed than cell invasion. This study demonstrated the survival and colonization ability of *E. coli* strains resistant to spectrum cephalosporin-resistant.

Krüger et al. discovered that the presence of mobile genetic elements encoding resistance and virulence genes results in variations in gene expression. Multiple drug resistance may be related to the existence of mobile genetic elements. *Salmonella* Infantis and *Salmonella* Heidelberg serotypes showing multiple drug resistance have been found to carry the *bla*_TEM − Ib_ and *bla*_CTX − M−65_ genes, which are related to mobile genetic elements.

The research conducted by Ma et al. investigated the prevalence of *Arcobacter* spp. in Shenzhen, China, as well as identifying its virulence and AMR through whole genome sequencing (WGS). This study discovered that antimicrobial resistant gene counts varied between different strains, and certain strains carried multiple drug resistance genes. Additionally, it was found that corresponding drug-resistant strains exhibited specific drug-resistance genes.

Finally, the results of Wang et al.'s study showed that the phage PaVOA effectively kills *Pseudomonas aeruginosa* within a short period using a rabbit skin infection model from New Zealand. Therefore, phage cocktail therapy represents a new approach to treating traumatic skin infections caused by MDR *Pseudomonas aeruginosa*.

In summary, this article Research Topic provides valuable insights into the epidemiological distribution and antimicrobial resistance of foodborne pathogens across various nodes of the food supply chain, aiding readers in understanding this important topic. This Research Topic of articles has garnered considerable attention from the appropriate practitioners, with over 11,000 views and a total of 2,315 downloads.

## Author contributions

YW wrote the initial draft of the manuscript while JZ, ZP, XW, and PP provided substantial critical feedback and editing. All authors contributed to the article and approved the submitted version.
